# Supplementary figures and images for: Stochasticity in dietary restriction-mediated lifespan outcomes in Drosophila
Source: GeroScience. 2025 Jan 31;47(3):4697–709. doi: 10.1007/s11357-025-01537-5 (PMC12181480; doi:10.1007/s11357-025-01537-5)

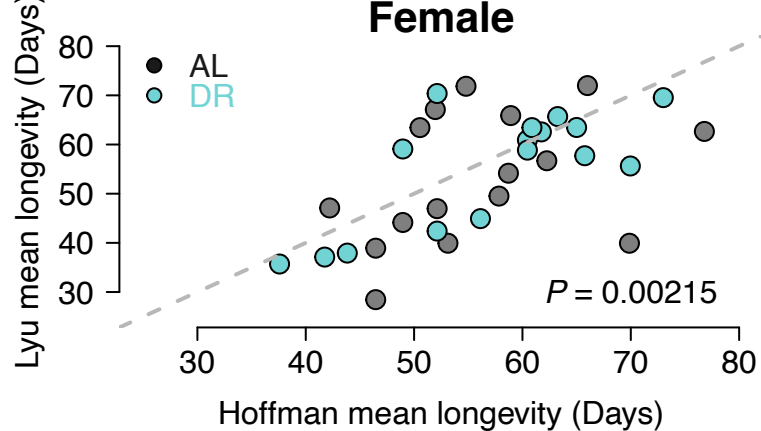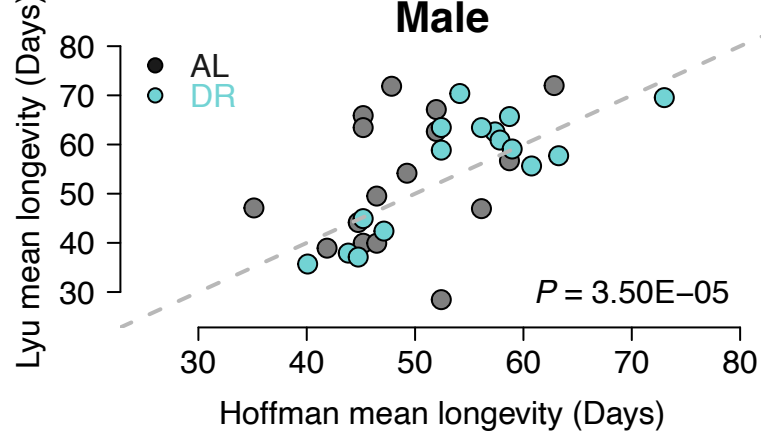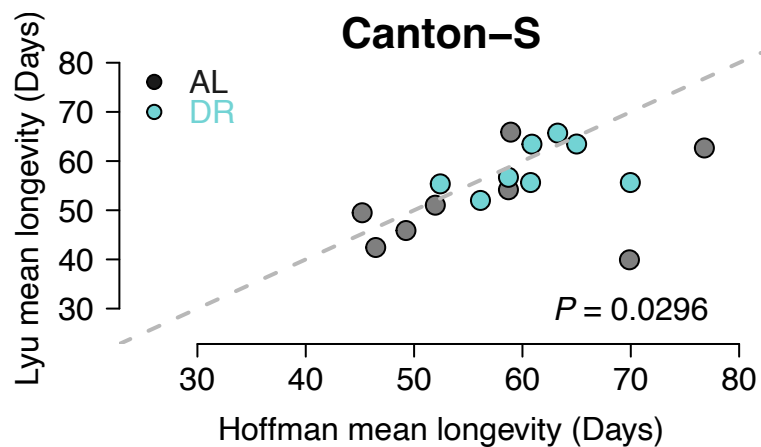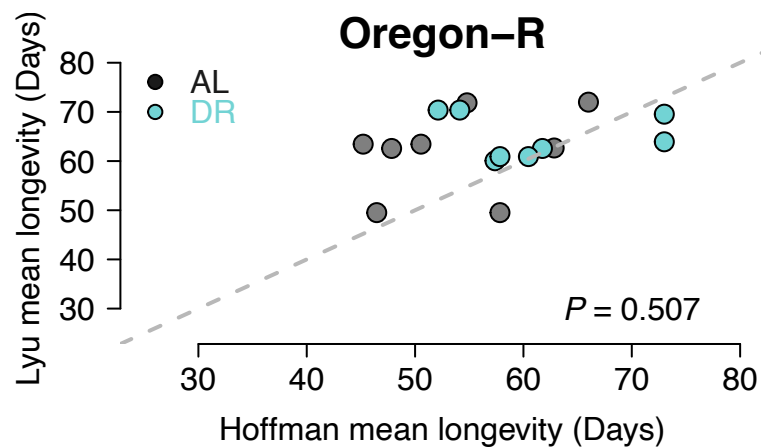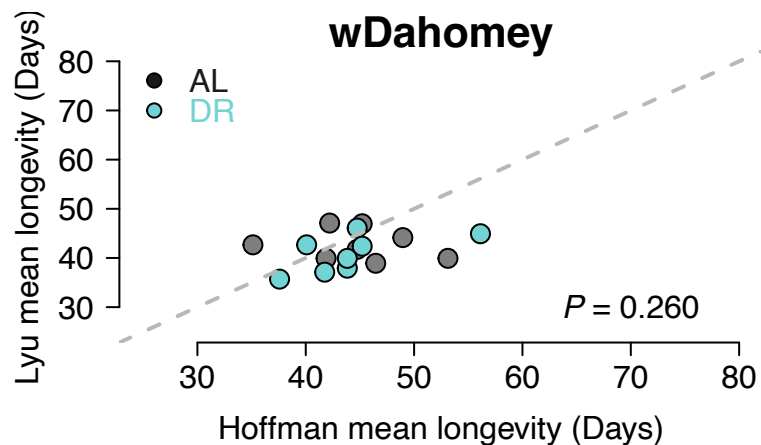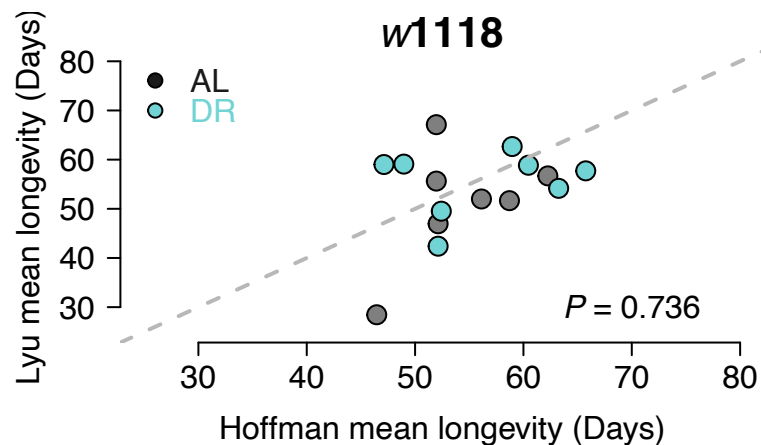

Supplement: Supplementary file 1 — Supplementary file1 Fig. 1 Correlation plot of cohort pairs stratified by genotype between the two labs. Each point represents the mean longevity for the Hoffman lab (x-axis) and the Lyu lab (y-axis). The black line represents the line of symmetry. P values are derived from the Spearman correlation test (PDF 229 kb) [file 11357_2025_1537_MOESM1_ESM.pdf]

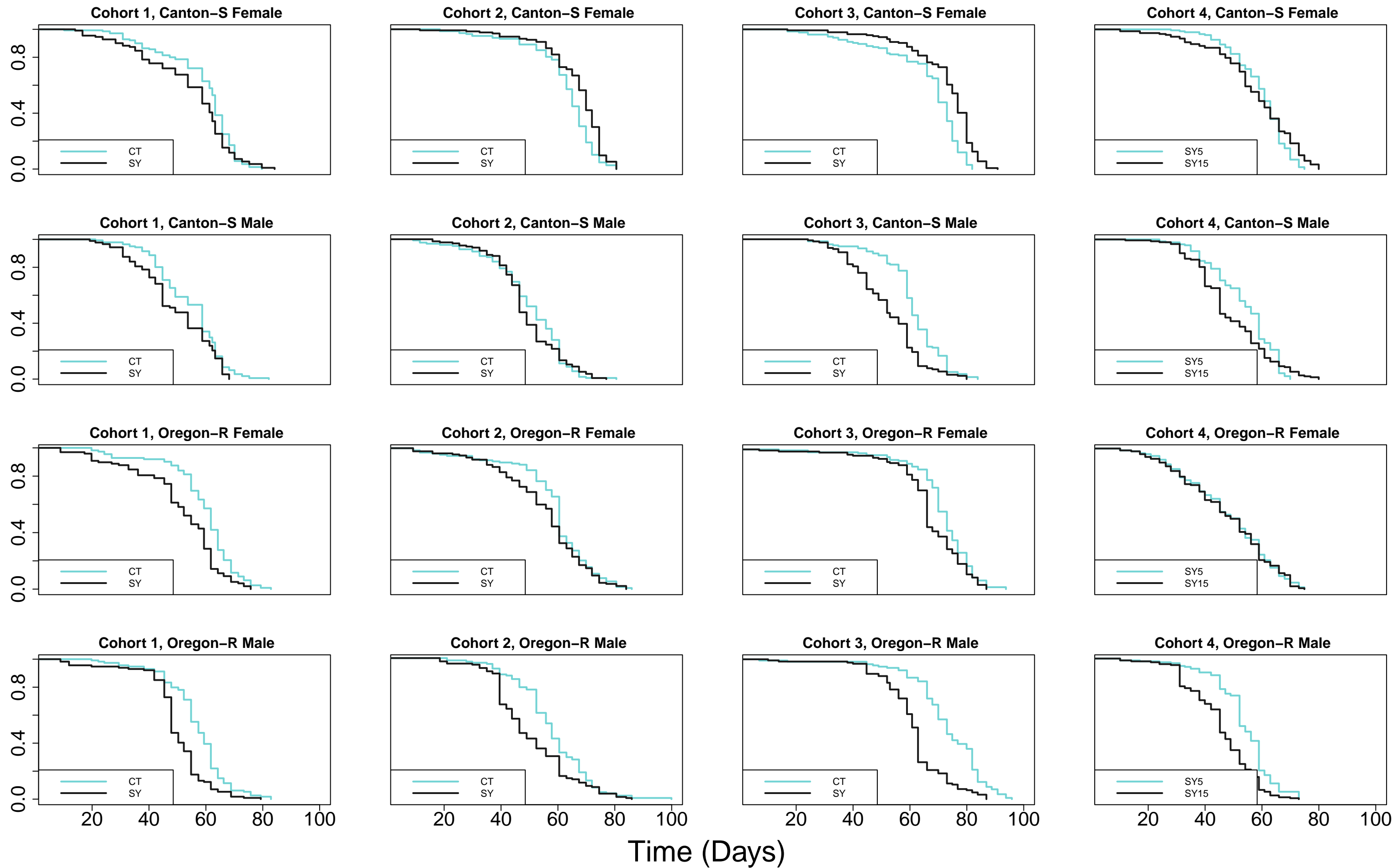

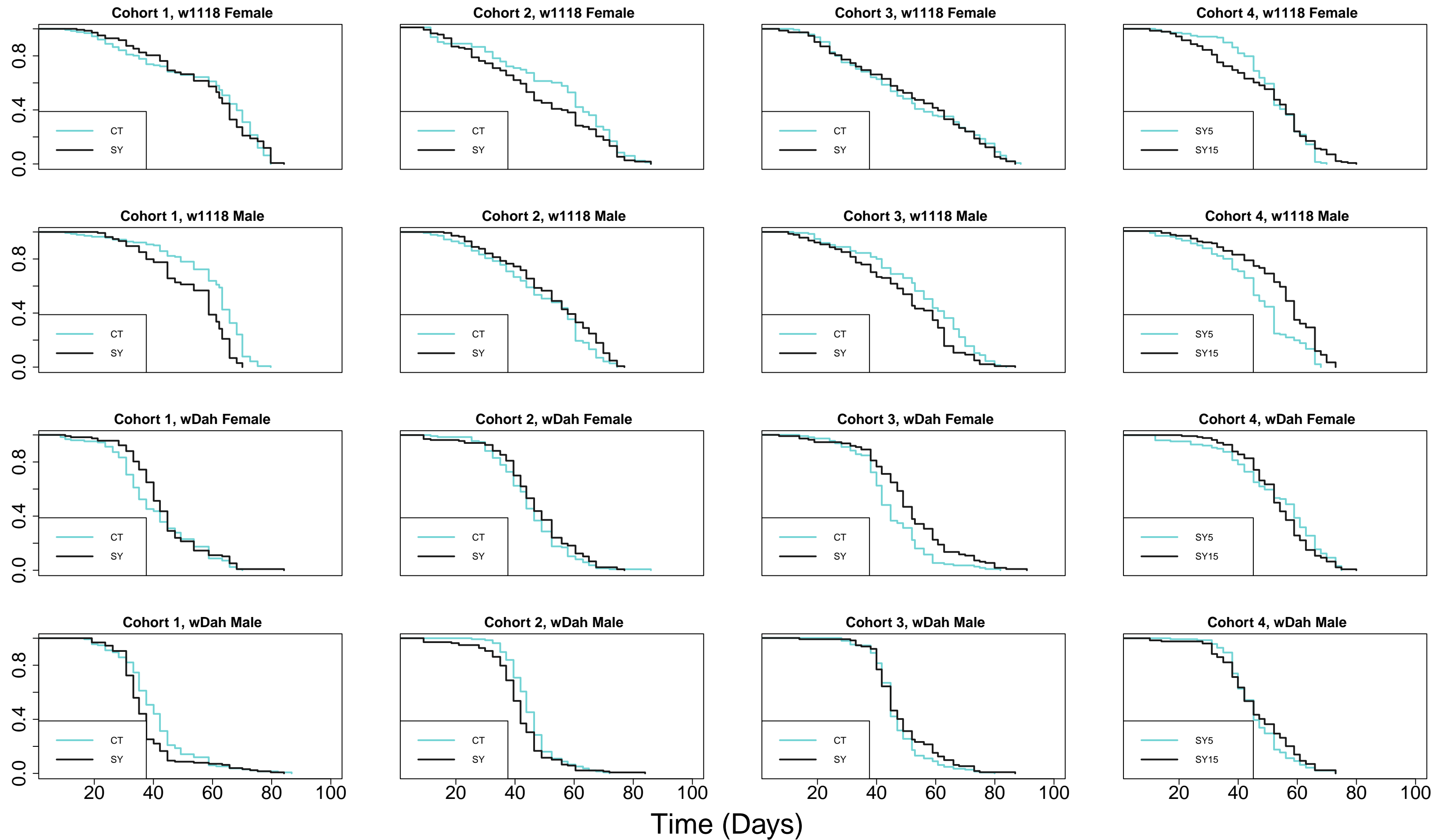

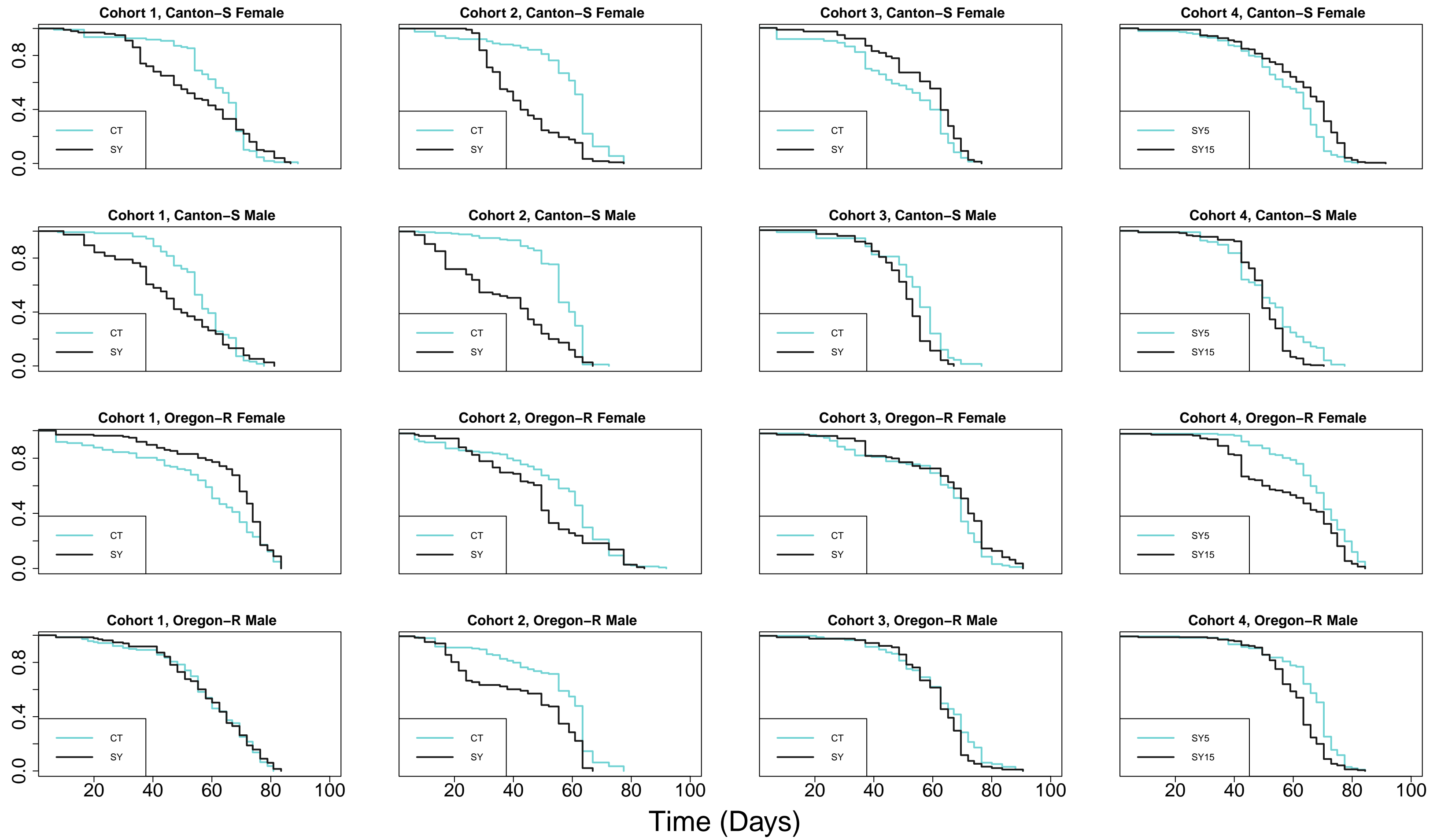

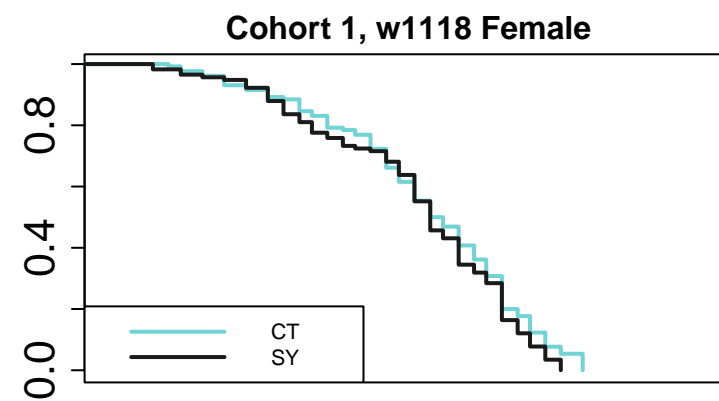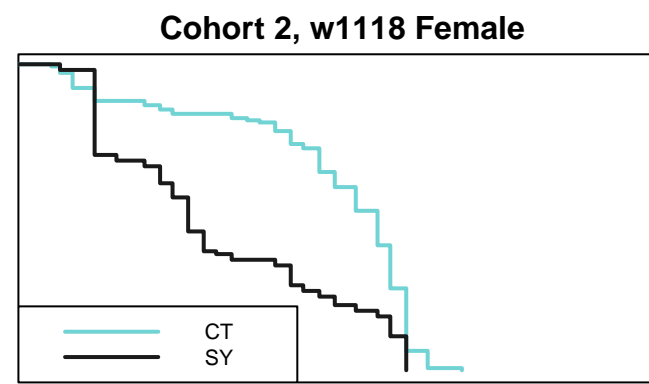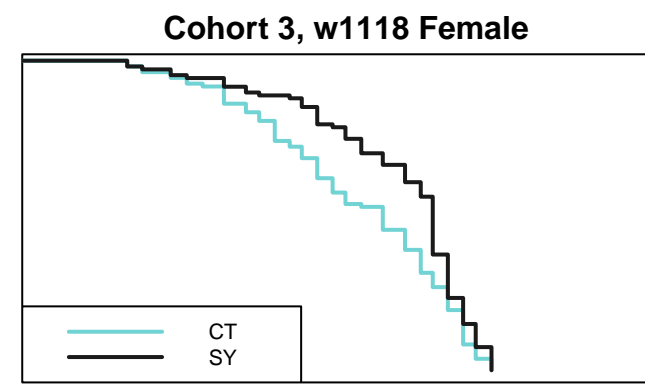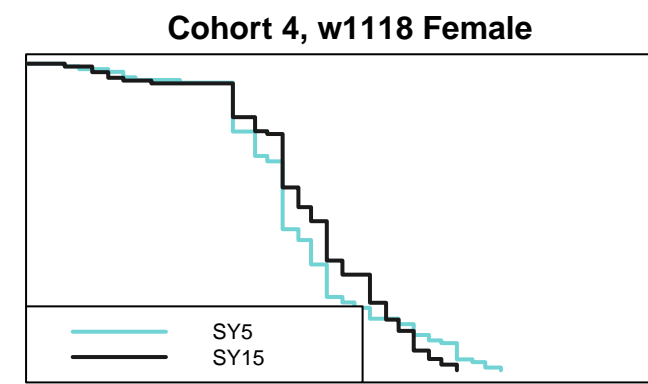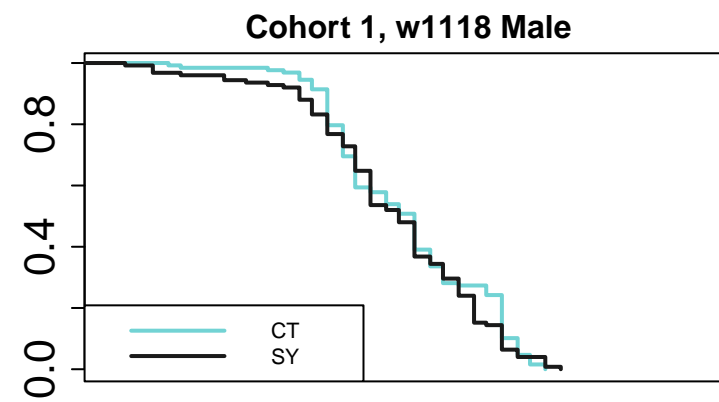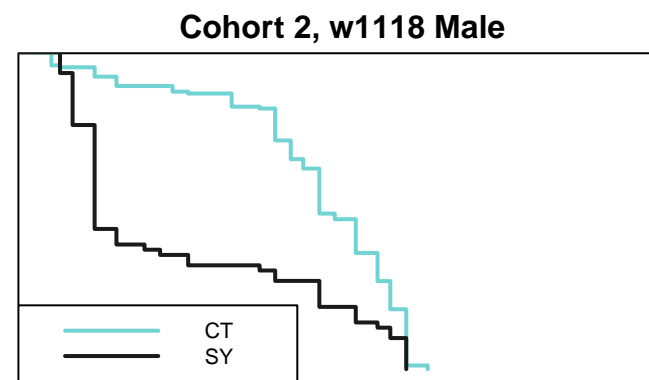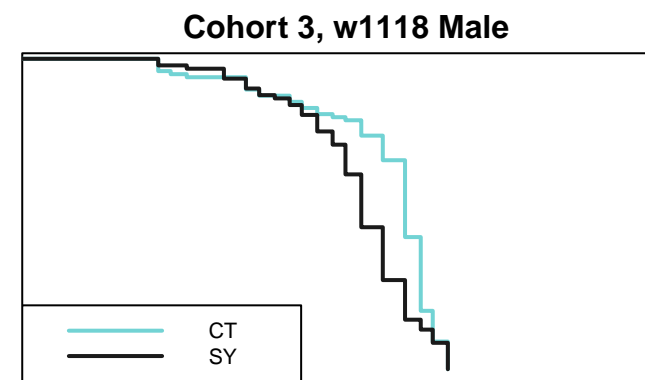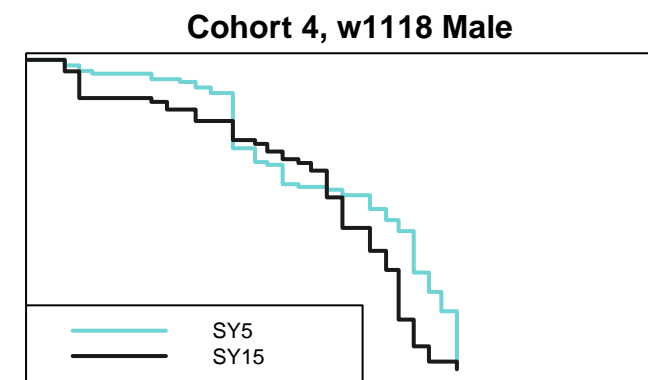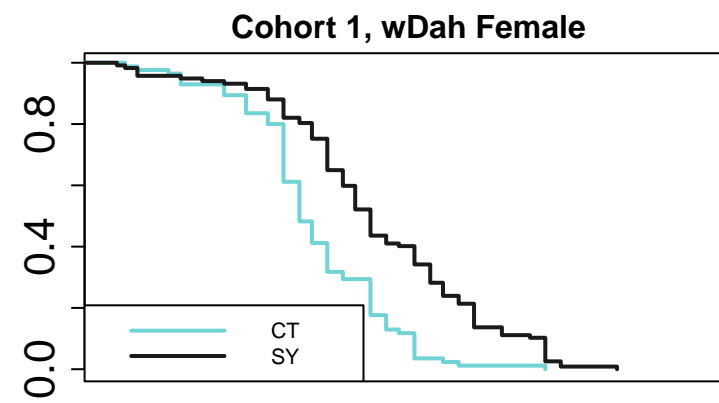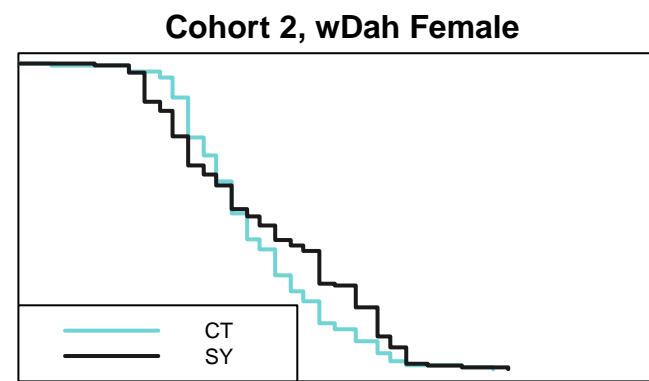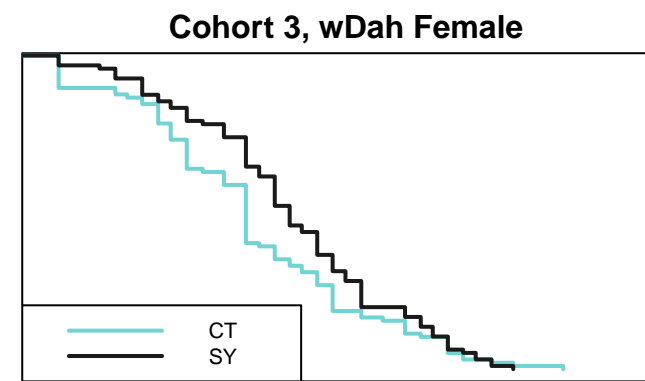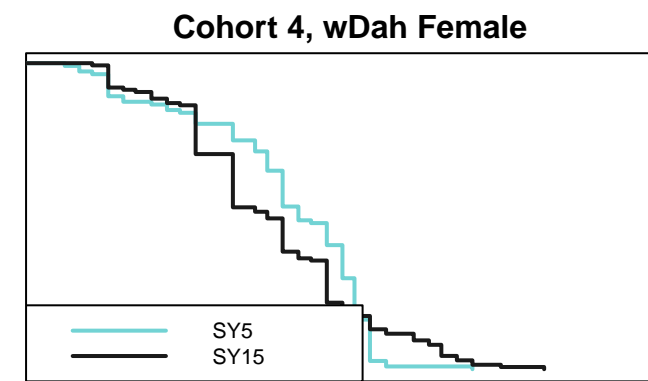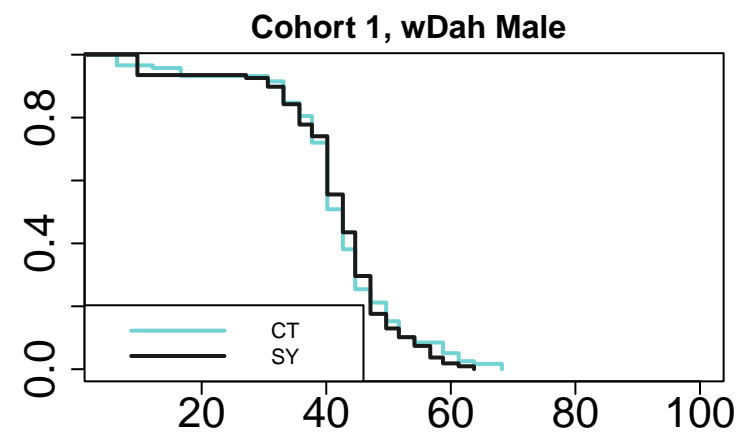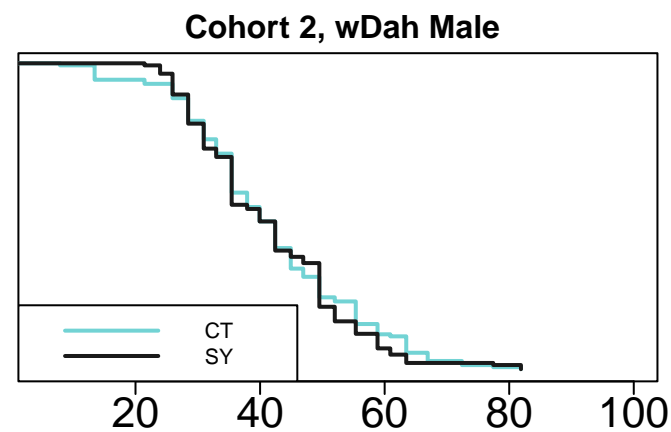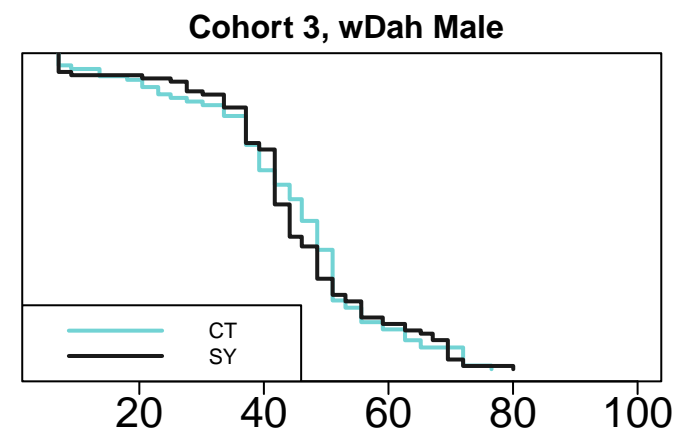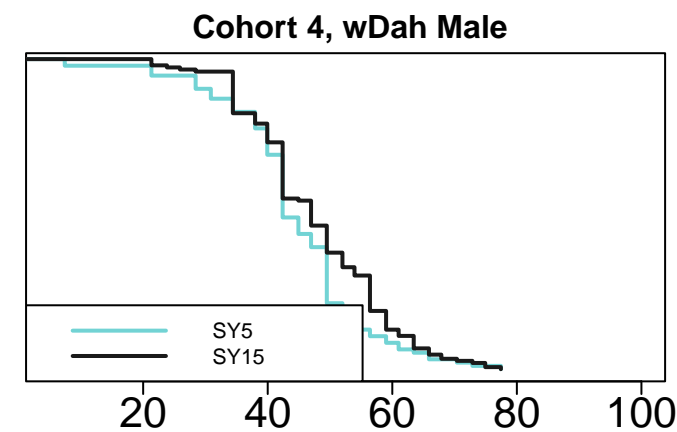

Time (Days)

Supplement: Supplementary file 2 — Supplementary file2 Fig. 2 Kaplan–Meier curves of each of 64 pairs of AL/DR experiments (PDF 43 kb) [file 11357_2025_1537_MOESM2_ESM.pdf]

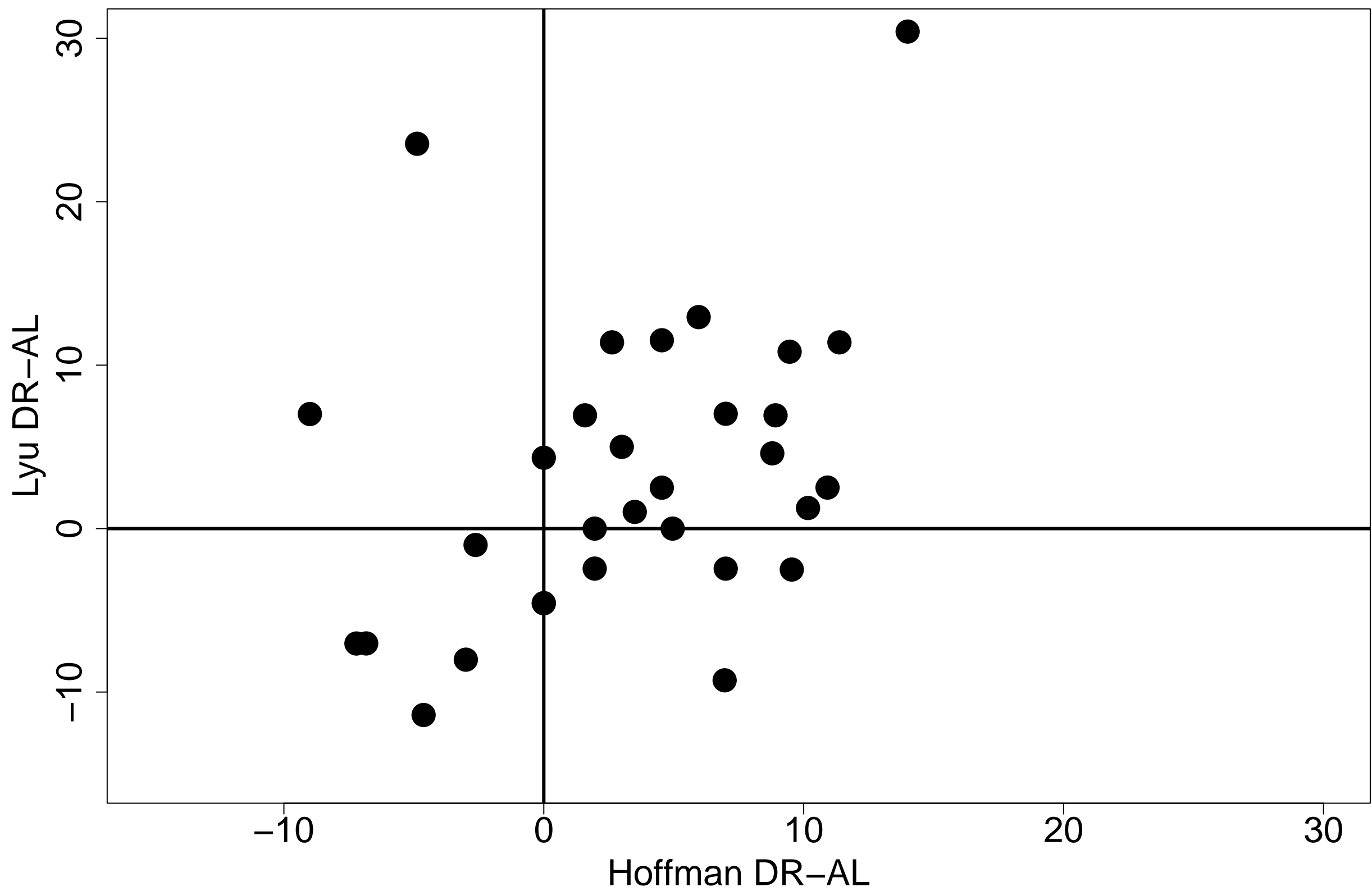

Supplement: Supplementary file 3 — Supplementary file3 Fig. 3 Difference of AL and DR median lifespan between labs (PDF 6.22 kb) [file 11357_2025_1537_MOESM3_ESM.pdf]

Treatment CT SY

A  
Female

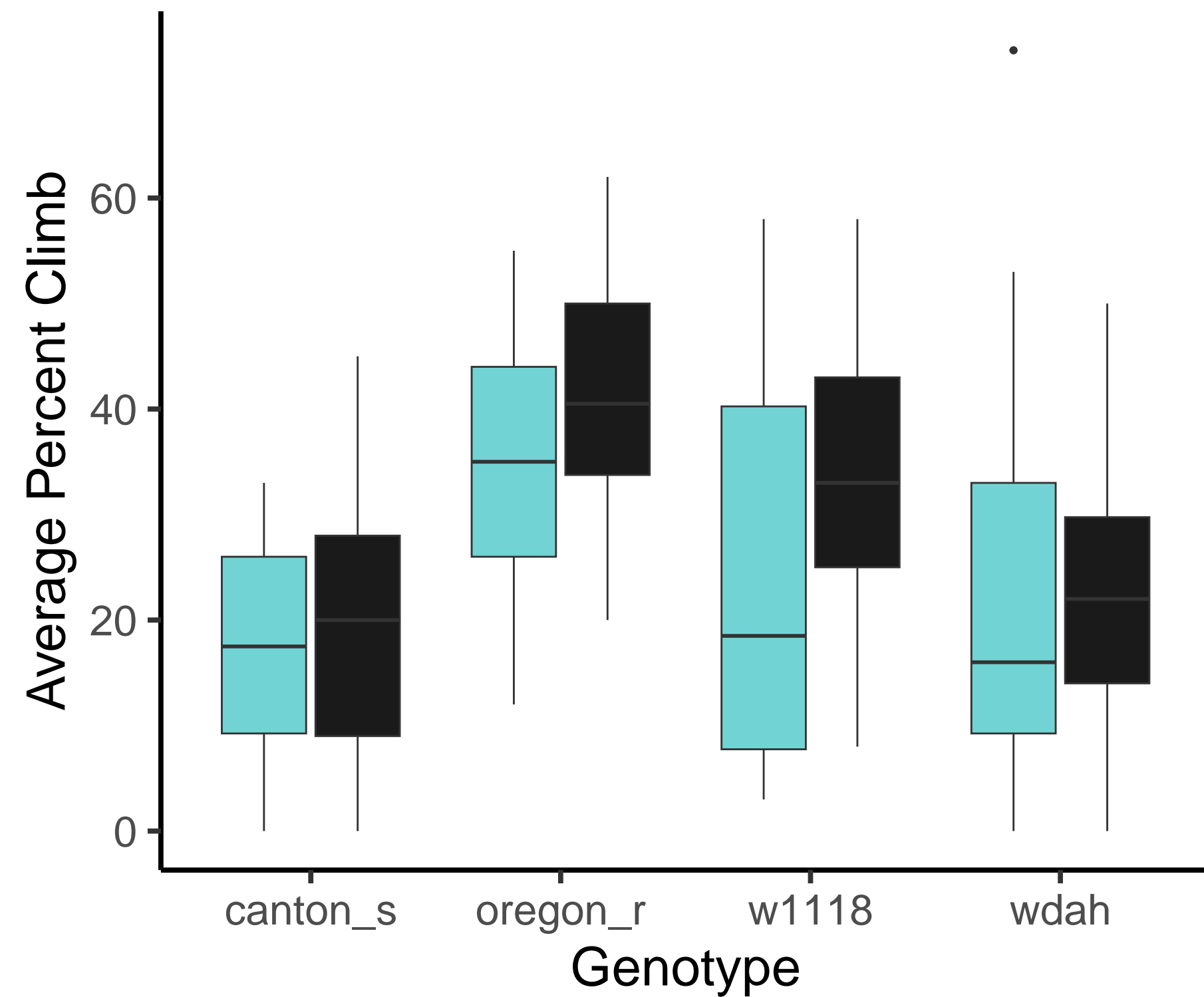

B  
Male

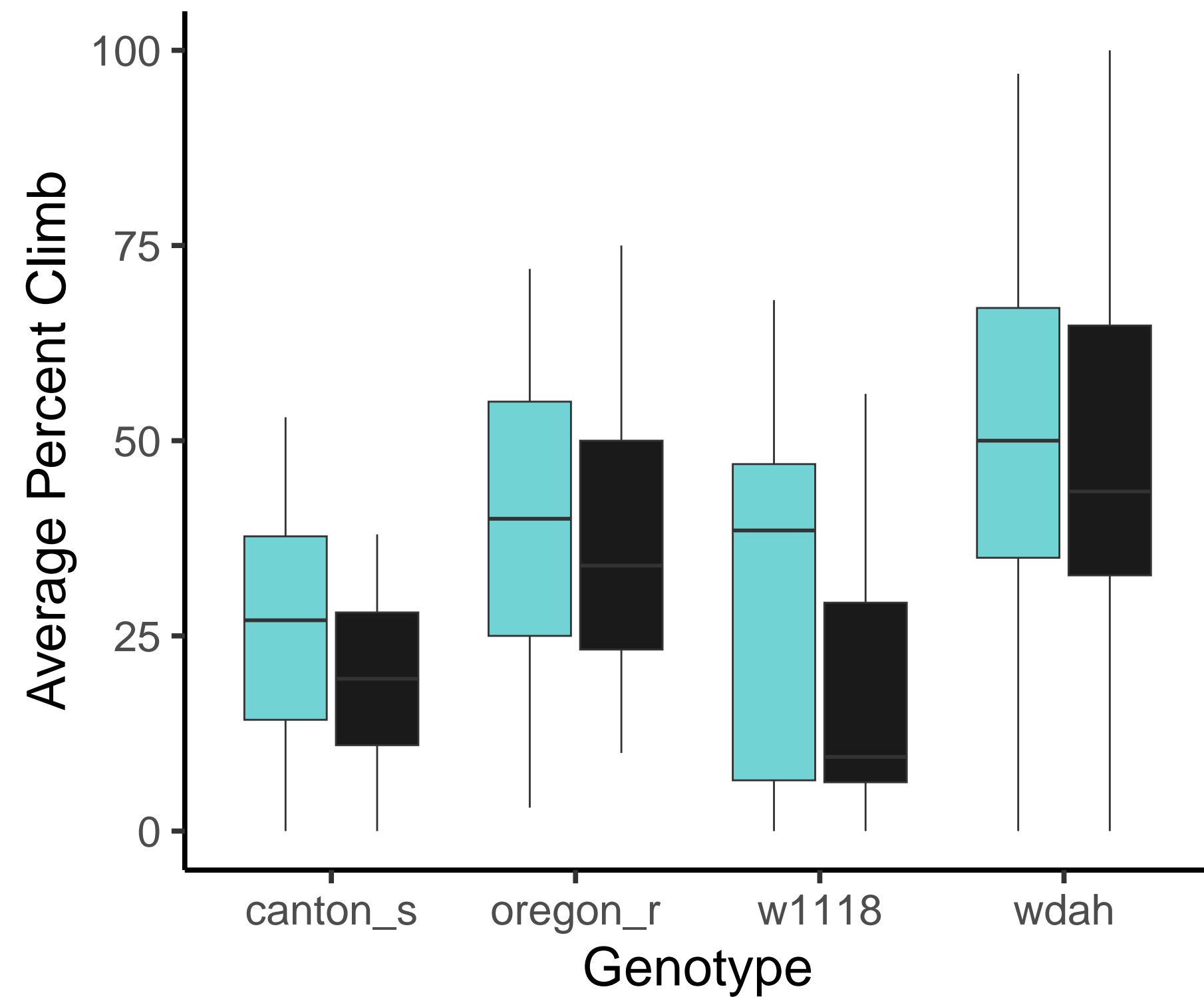

Supplement: Supplementary file 4 — Supplementary file4 Fig. 4 Climbing results for 30-day-old flies from the Hoffman lab for females (A) and males (B). Each replicate consists of 18 vials of ~20 flies each. Mean climbing values were taken on a per vial average. Cohorts 1–3 were combined for analysis. There were significant effects of sex and genotype with no difference between AL and DR treatments (PDF 6 kb) [file 11357_2025_1537_MOESM4_ESM.pdf]

Treatment ■ SY5 ■ SY15

**A**  
**Female**

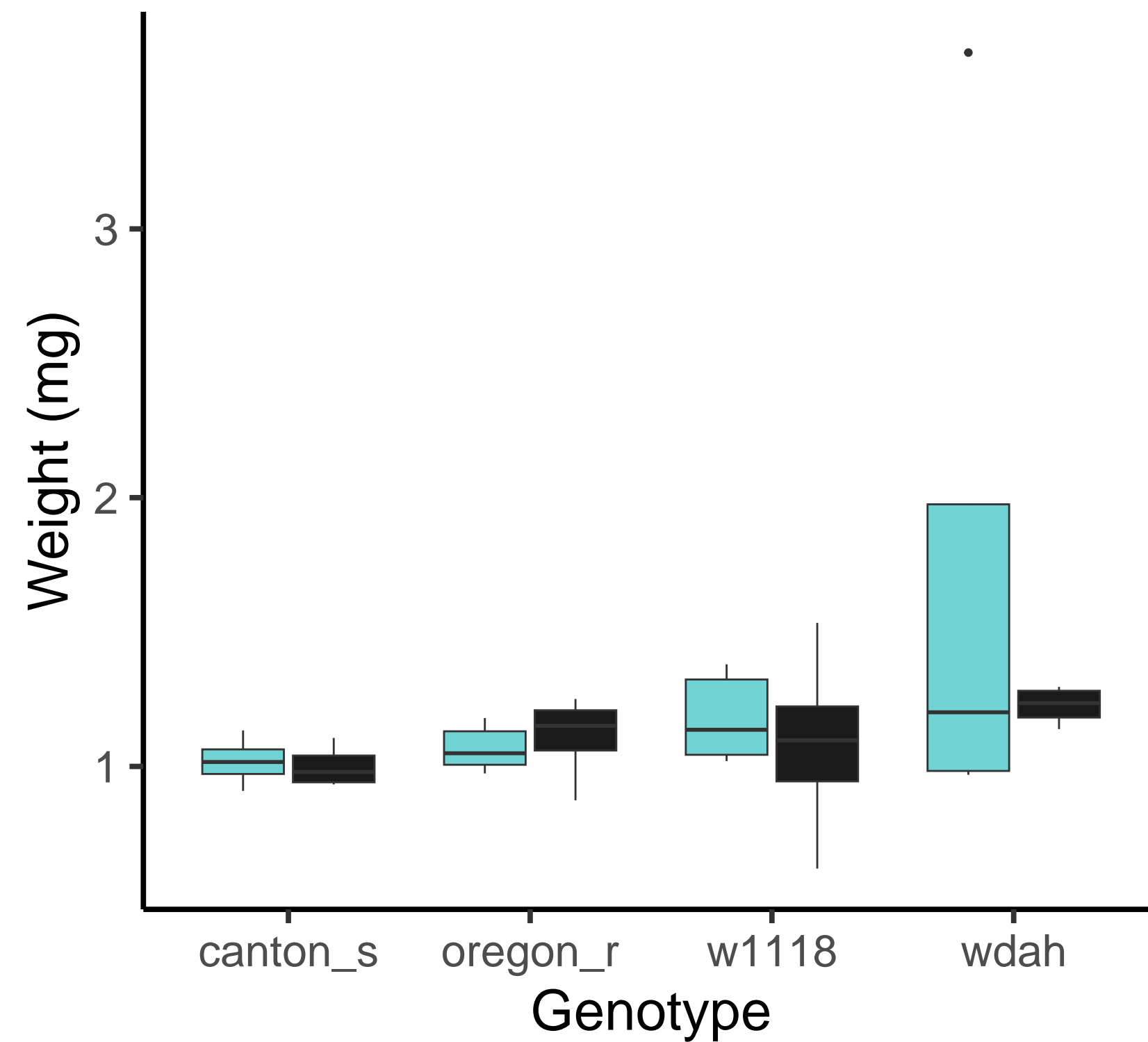

**B**  
**Male**

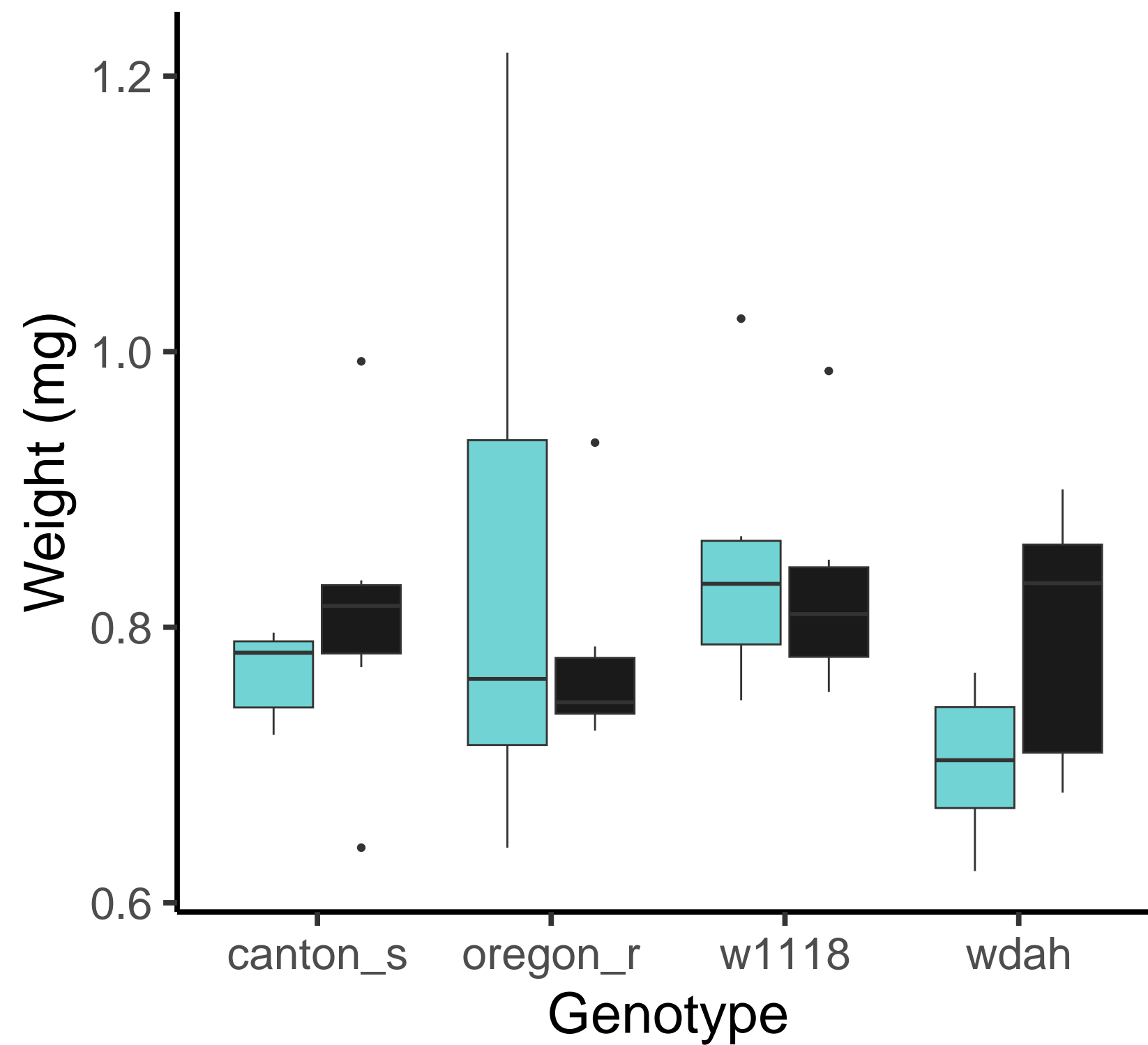

Supplement: Supplementary file 5 — Supplementary file5 Fig. 5 Body mass results for 30-day-old flies on SY5 vs SY15 for females (A) and males (B). Each replicate consisted of ~ 5 measurements of 5 flies each. There were no significant effects of treatment, suggesting that our flies were not calorically restriction on the DR treatment. Females were significantly larger than males as expected, and no genotype effects were seen (PDF 6 kb) [file 11357_2025_1537_MOESM5_ESM.pdf]

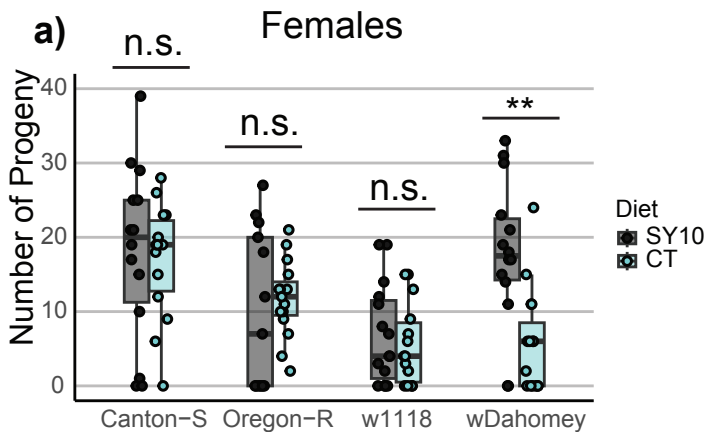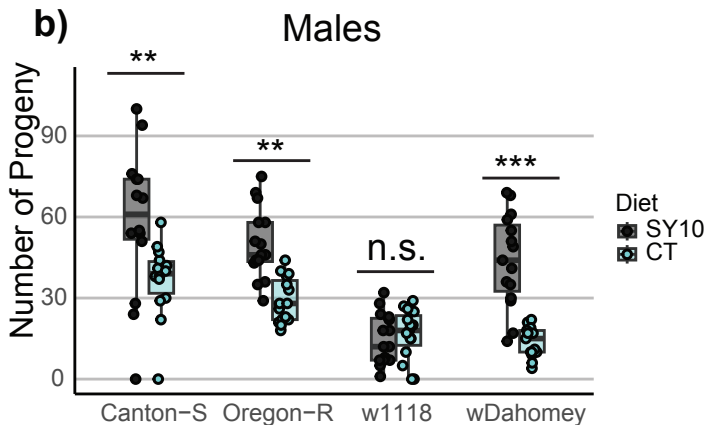

Supplement: Supplementary file 6 — Supplementary file6 Fig. 6 Dietary effects on fertility in 7-day-old females (A) and males (B). Each replicate consisted of one targeted fly and two flies of the opposite sex. Significant dietary effects were observed in both sexes, with males showing stronger effects. Adjusted P values from Tukey’s HSD test are calculated for each genotype. Statistical significance is denoted as follows: n.s. (not significant) > 0.05; ** < 0.01; *** < 0.001 (PDF 508 kb) [file 11357_2025_1537_MOESM6_ESM.pdf]
