# Supplementary material for: Stochasticity in dietary restriction-mediated lifespan outcomes in Drosophila
Source: GeroScience. 2025 Jan 31;47(3):4697–709. doi: 10.1007/s11357-025-01537-5 (PMC12181480; doi:10.1007/s11357-025-01537-5)
Supplement: Supplementary file 8 — Supplementary file8 (PDF 35 kb) [file 11357_2025_1537_MOESM8_ESM.pdf]

Supplementary Table 2. Cox-Snell R^2 Values for full and reduced models to assess individual covariate contributions.

Factor	Cox-Snell R^2 (Cohort 1-4)
Full model	0.1963838343
Reduced model (-Lab)	0.1927282311
Reduced model (-Cohort)	0.1670675846
Reduced model (-Diet)	0.1951061488
Reduced model (-Sex)	0.1748700783
Reduced model (-Genotype)	0.0735367406
