# Supplementary material for: Stochasticity in dietary restriction-mediated lifespan outcomes in Drosophila
Source: GeroScience. 2025 Jan 31;47(3):4697–709. doi: 10.1007/s11357-025-01537-5 (PMC12181480; doi:10.1007/s11357-025-01537-5)
Supplement: Supplementary file 9 — Supplementary file9 (PDF 10 kb) [file 11357_2025_1537_MOESM9_ESM.pdf]

Supplementary Table 3. Variance analysis using our likelihood-based method, presented separately for each genotype.

	w[Dahomey]	*w*[1118]	Canton-S	Oregon-R
Lab	10.93%	22.60%	4.28%	21.84%
Cohort	48.88%	53.57%	15.07%	56.12%
Diet	16.95%	7.79%	2.95%	11.49%
Sex	23.24%	16.03%	77.70%	10.54%
